# Additional cleft mitral valve diagnosed by a combination of 2-D and 3-D echocardiography using transesophageal echocardiography during mitral valve prolapse: a case report

**DOI:** 10.1186/s40981-020-00337-4

**Published:** 2020-05-01

**Authors:** Kazuto Miyata, Sayaka Shigematsu

**Affiliations:** Department of Anesthesia, New Heart Watanabe Institute, Hamadayama 3-19-11, Suginami-ku, Tokyo, 168-0065 Japan

**Keywords:** Mitral valve prolapse, Cleft mitral valve, Mitral valve repair

## Abstract

**Background:**

A mitral cleft may be an important etiological factor for significant mitral regurgitation. We diagnose an additional cleft mitral valve by a combination of 2-dimensional (2-D) and 3-dimensional (3-D) echocardiography.

**Case presentation:**

We describe the case of a severe mitral regurgitation due to posterior leaflet prolapse (P2). In the 2-D view, which is obtained after turning the probe clockwise from the mid-esophageal long-axis view, TEE showed a moderate central regurgitation jet. In the 3-D en face view, a cleft between P2 and P3 was identified, and we found that the cause of mitral regurgitation was not only P2 prolapse but also a cleft between P2 and P3.

**Conclusion:**

A complex mitral valve lesion was detected by a combination of 2-D and 3-D TEE. The presence of a cleft could affect the surgical procedure because of the possibility that an enlarged cleft would increase with leaflet resection.

## Background

Cleft mitral valve is a rare finding in adult cardiovascular medicine. The accurate diagnosis of complex mitral valve lesions is important for mitral valve repair [1]. However, no standard method has been proposed for diagnosing the etiology of mitral regurgitation, especially a cleft, by transesophageal echocardiography (TEE).

## Case presentation

A 51-year-old man (height 168 cm; weight 57 kg) had severe mitral regurgitation due to posterior leaflet prolapse (P2) with exertional dyspnea. He had normal systolic function (ejection fraction 75%) with no past history. He was scheduled to undergo robot-assisted mitral valve repair.

General anesthesia was induced with 5 mg of midazolam, 50 mg of rocuronium, and 0.5 μg/kg/min of remifentanil intravenously. The trachea was intubated with a left-sided 37-French (Fr) double-lumen tube, followed by an insertion of a TEE probe CX-50 TEE machine (Philips Medical Systems Andover, MA). A central venous catheter and pulmonary artery catheter were placed in the left internal jugular vein, and a 16-Fr venous cannula was inserted through the right internal jugular vein for drainage of the superior vena cava, with the tip at the junction of the superior vena cava and innominate vein. Anesthesia was maintained with 1.5% of sevoflurane in oxygen, continuous infusions of 0.2‑0.4 μg/kg/min of remifentanil, and 4 mg/kg/h of propofol.

**Fig. 1:**
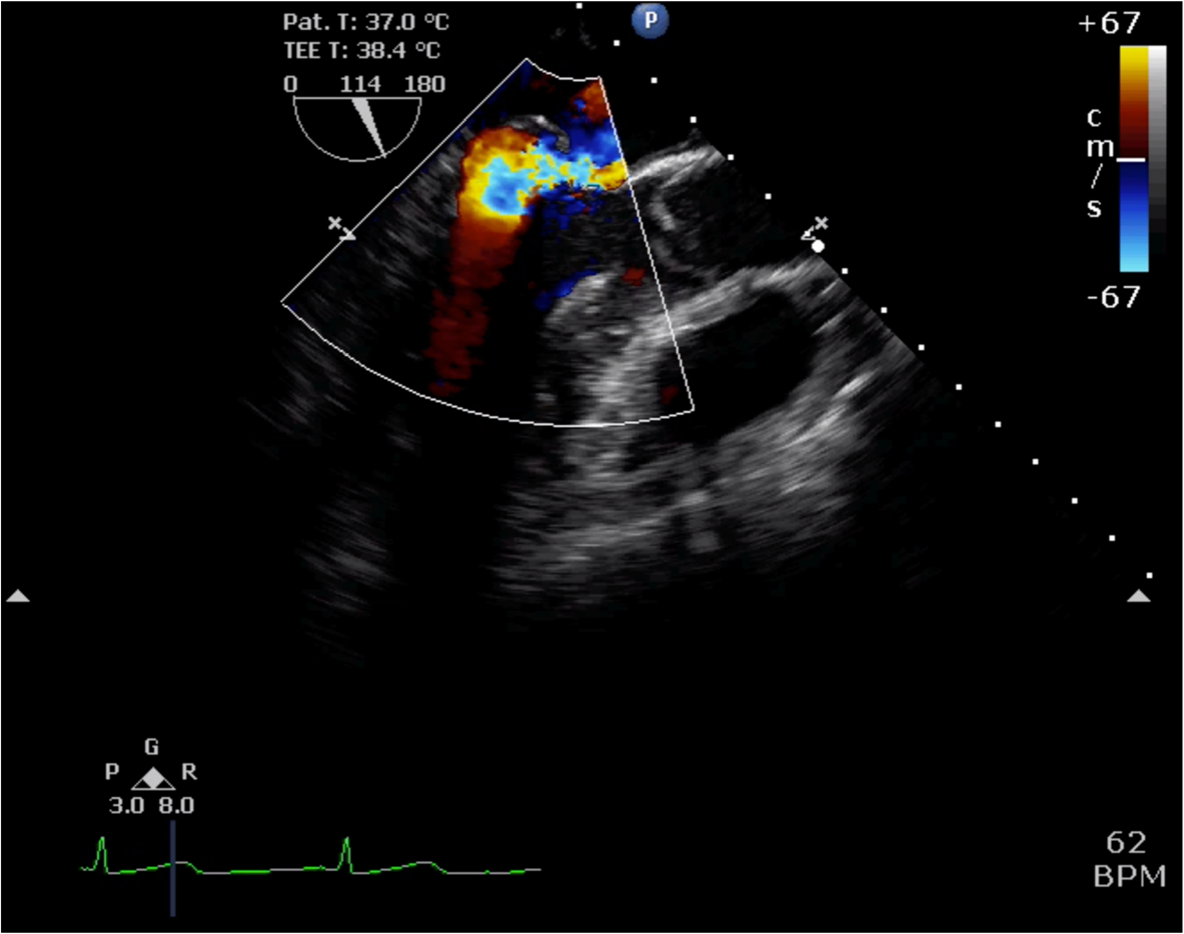
Mid-esophageal long-axis view showing eccentric mitral valve regurgitation due to P2 prolapse

**Fig. 2:**
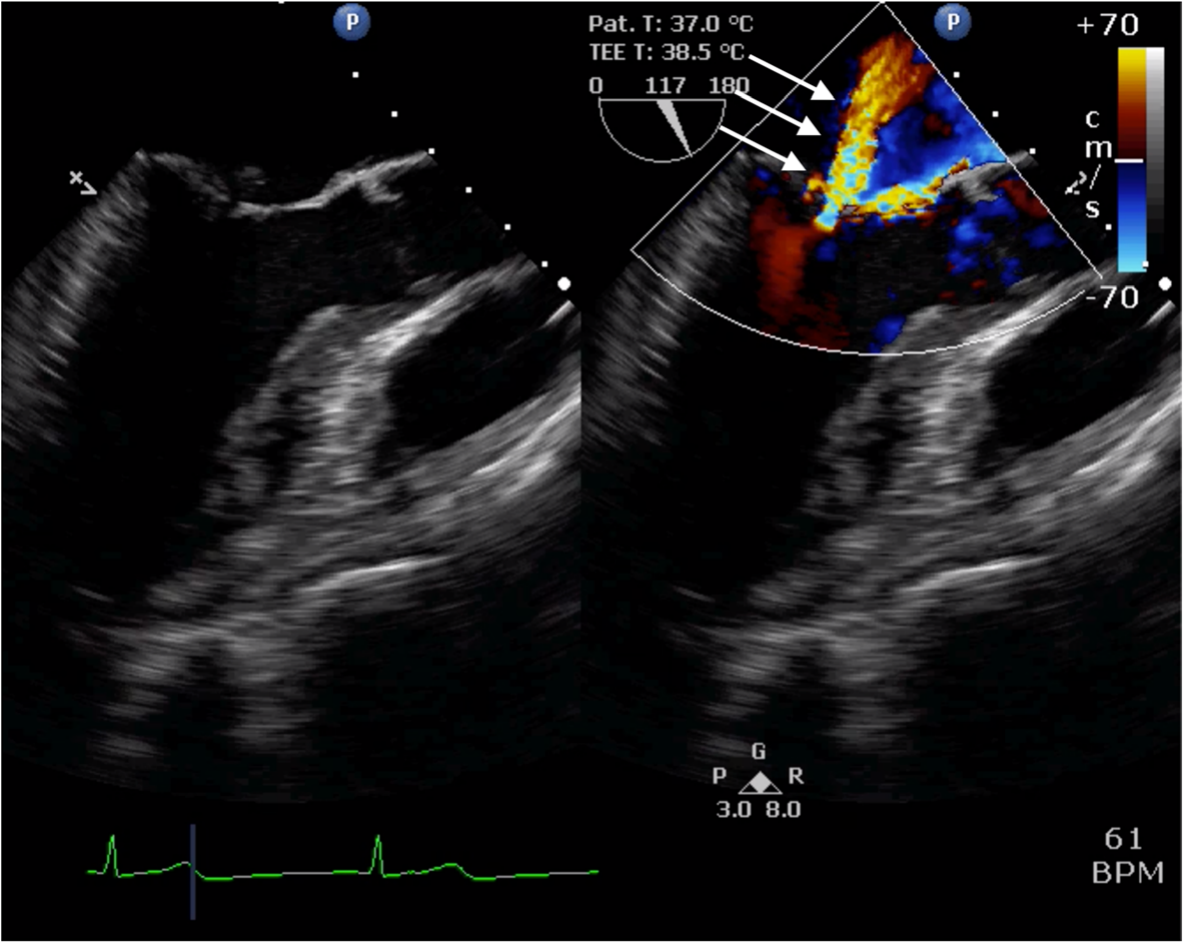
Clockwise rotation of probe in the mid-esophageal long-axis view shows normal configuration of the mitral leaflets (A3-P3), however, a color Doppler image reveals a central mitral valve regurgitation jet with unknown etiology (white arrow)

**Fig. 3:**
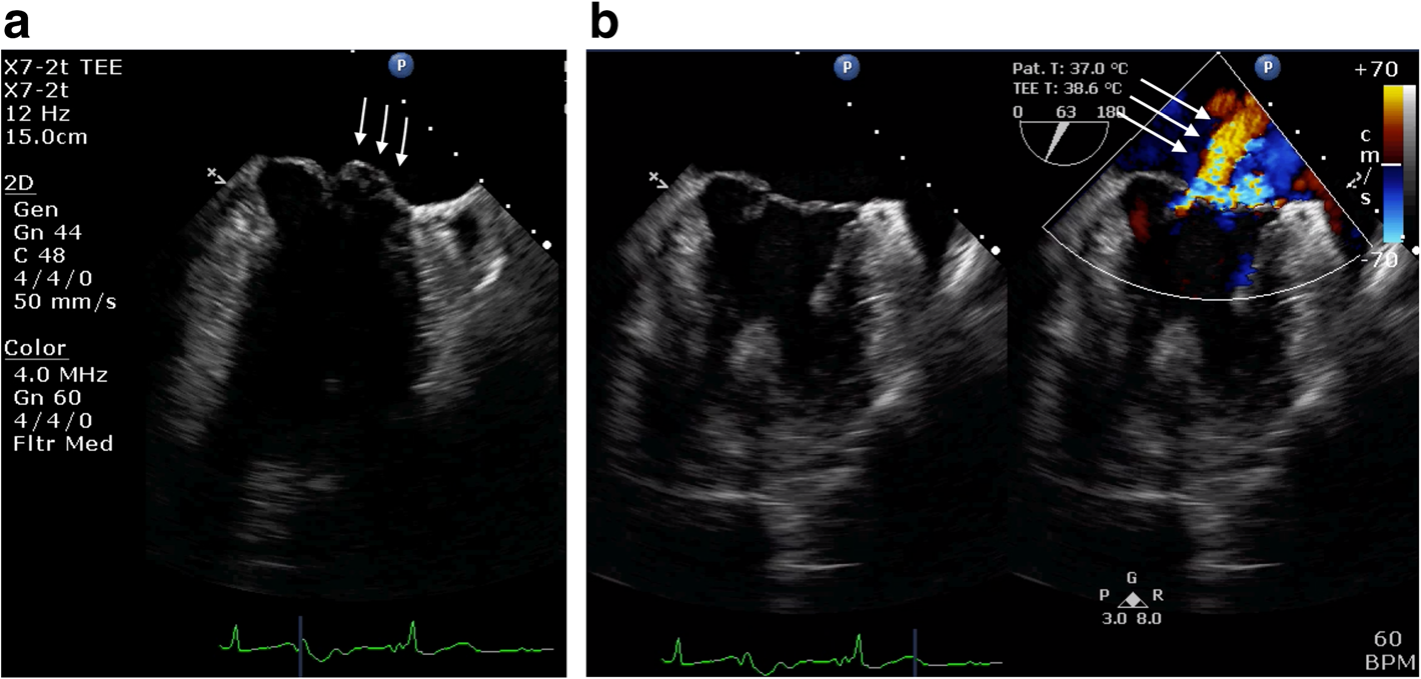
**a** Mid-esophageal commissure view showing a “cobra head sign” indicating P2 prolapse (white arrow). **b** Mid-esophageal commissure view showing a central mitral valve regurgitation jet with unknown etiology between A3 and P3 (white arrow)

TEE showed posterior leaflet prolapse (P2) with a marked eccentric jet in the mid-esophageal long-axis view (Fig. 1). After turning the probe clockwise from the mid-esophageal long-axis view, TEE showed a moderate central regurgitation jet (Fig. 2). In the mid-esophageal commissure view, the “cobra head sign” indicating P2 prolapse (Fig. 3a) was noted. Moreover, a central regurgitation jet that was induced between the anterior leaflet (A3) and posterior leaflet (P3) was observed (Fig. 3b).

**Fig. 4:**
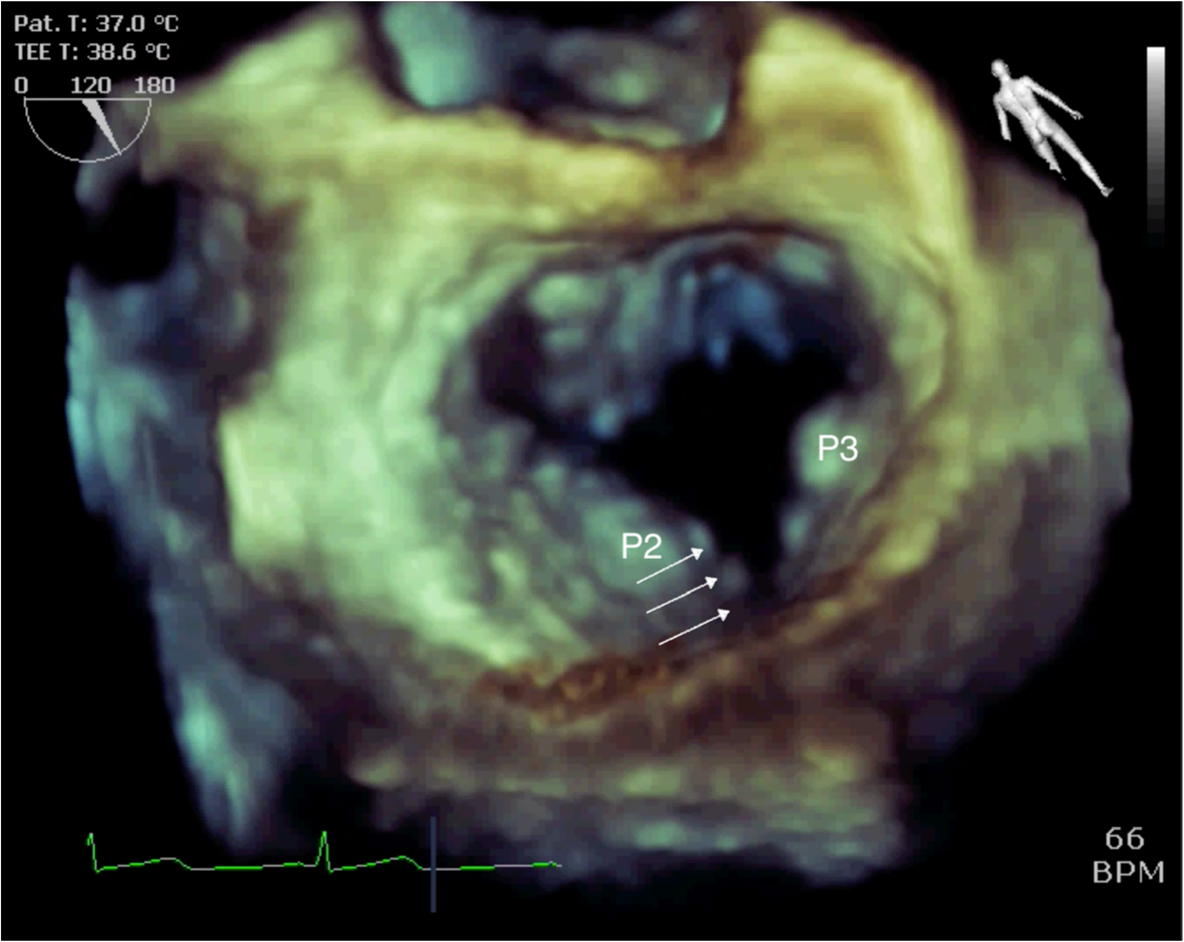
Three-dimensional echocardiography at the en face view at early diastole showing a profound indentation (cleft) between P2 and P3 (white arrow)

The three-dimensional (3-D) en face view showed a profound indentation, indicating that the cleft between P2 and P3 was noted at early diastole (Fig. 4). TEE showed not only P2 prolapse but also a cleft between P2 and P3, and mitral regurgitation was caused by both etiologies.

After establishing cardiopulmonary bypass (CPB), an antegrade cardioplegia cannula was inserted into the ascending aorta and a flexible cross-clamp was subsequently applied.

For the mitral valve repair procedure, posterior leaflet resection was not performed; instead, neochordal reconstruction of P2 and P3 and ring annuloplasty were performed due to the risk of cleft dilation after resecting the posterior leaflet. After declamping of the aorta, a 3 μg/kg/min dopamine infusion was started. Weaning from CPB was very smooth. However, systolic anterior motion (SAM) occurred immediately after weaning from cardiopulmonary bypass. Dopamine was stopped to administrate. After volume loading and beta-blocker administration, SAM was improved. The hemodynamic status was stable. Subsequently, residual mitral regurgitation was not observed, and the postoperative course was uneventful.

## Discussion

We found out two important clinical issues: firstly, intraoperative TEE, which is a combination of 2-D and 3-D echocardiography, was useful for diagnosing mitral regurgitation due to a cleft between P2 and P3 with P2 prolapse. Secondly, it was more useful for the selection of the mitral valve repair procedure by diagnosing the complicated mitral valve lesion which recognized the mitral regurgitation from the cleft.

After turning the probe clockwise from the mid-esophageal long-axis view, which indicates the modified mid-esophageal long-axis view, the view revealed the medial (A3-P3) side of the mitral valve. In the mid-esophageal commissure view, P3 and opposite site, which indicates not A2 but A3, was revealed. After turning the probe clockwise, a central mitral regurgitation jet was observed, although no prolapse was noted in the valve leaflets. In the mid-esophageal commissure view, a prolapse of A3 and P3 was not observed, but a central mitral regurgitation jet was observed. Finally, the 3-D en face view showed profound indentation in the mitral annulus indicating a cleft between P2 and P3. These results suggest that the central mitral regurgitation jet originated from the cleft of the posterior leaflet between P2 and P3.

In this case, mitral regurgitation was caused by the cleft between P2-3 in addition to the P2 prolapse. It also affected the selection of the operative method. For P2 prolapse, surgical resection of P2 is usually the choice for mitral valve repair. However, one cause of residual mitral regurgitation after mitral valve repair may be enlarged clefts. Therefore, in the case of mitral leaflet prolapse in conjunction with a cleft, there was a possibility that the risk of an enlarged cleft would increase with leaflet resection. Moreover, a cleft is a potential source of residual mitral regurgitation.

According to many reports, the frequency of mitral valve cleft is low. In a study using 2-D TEE, only 0.07% of patients with moderate or greater mitral regurgitation had a cleft on the posterior leaflet [2]. However, technological advances led to the emergence of 3-D echocardiography, and guidelines recommend that the mechanism of mitral regurgitation may be determined through the routine use of 3-D echocardiography [3]. In a study, 3-D echocardiography revealed the existence of a cleft in 3.3% of patients with moderate or greater mitral regurgitation [4]; 2.5% of these clefts were in the anterior leaflet and 0.8% in the posterior leaflet [4]. Thus, even with 3-D echocardiography, clefts in the posterior leaflet are rarely noted. This case is one of complex mitral valve lesions which indicates P2 prolapse and a cleft between P2 and P3.

In conclusion, we found that a complicated mitral valve lesion including P2 prolapse and a cleft between P2 and P3 was detected by 2-D and 3-D echocardiography using TEE. The presence or absence of mitral regurgitation from the cleft may also affect the choice of the mitral valve repair procedure.


**Additional file 1: Video S1.**



## Data Availability

Not applicable

## Abbreviations

TEE: Transesophageal echocardiography
